# Alcohol and Cannabis co-use and HIV risk, Treatment and Prevention Outcomes: A Scoping Review

**DOI:** 10.1007/s40429-025-00707-x

**Published:** 2026-01-28

**Authors:** Yen-Tyng Chen, Megan E. Marziali, Christopher Magana, Julia Anne Maxwell, Tammy Chung, Jimi Huh, Jade Pagkas-Bather, Justin Knox

**Affiliations:** 1 Edward J. Bloustein School of Planning and Public Policy, Rutgers, The State University of New Jersey, 33 Livingston Ave, Room 535, New Brunswick, NJ 08901, USA; 2 Department of Epidemiology, Mailman School of Public Health, Columbia University, New York, NY, USA; 3 New York State Psychiatric Institute, HIV Center for Clinical and Behavioral Studies, New York, NY, USA; 4 Department of Sociomedical Sciences, Mailman School of Public Health, Columbia University, New York, NY, USA; 5 Rutgers University, New Brunswick, NJ, USA; 6 Center for Population Behavioral Health, Institute for Health, Health Care Policy and Aging Research, Rutgers, The State University of New Jersey, New Brunswick, NJ, USA; 7 Department of Population & Public Health Sciences, Keck School of Medicine, University of Southern California, Los Angeles, CA, USA; 8 Chicago Center for HIV Elimination, University of Chicago, Chicago, IL, USA; 9 Department of Medicine, University of Chicago, Chicago, IL, USA

**Keywords:** Alcohol, Cannabis, Marijuana, HIV, Co-use, Simultaneous use

## Abstract

**Purpose of Review:**

Alcohol and cannabis are substances commonly used by people with or made vulnerable to HIV. With changing cannabis legalization, cannabis use has been on the rise, including simultaneous use (co-use) with alcohol. Prior reviews have assessed patterns, correlates, and consequences of alcohol and cannabis co-use. We conducted a scoping review to examine alcohol and cannabis co-use and HIV risk, treatment, and prevention outcomes.

**Recent Findings:**

We identified 818 unique articles across seven databases through December 2024, of which, 28 met criteria. There was substantial heterogeneity in the conceptualization of co-use, measurement, and analysis methods. Many studies employed cluster analyses, with alcohol and cannabis co-use often included in the context of polysubstance use. Only one study utilized event-level methods to assess simultaneous use and sex behaviors. Findings on the relationship between alcohol and cannabis co-use and antiretroviral medications (ARV) outcomes and sex behaviors are mixed, though several studies suggest that co-use may be associated with poorer ARV adherence and increased engagement in sex behaviors that increased HIV risks.

**Summary:**

This review highlights substantial heterogeneity in how alcohol and cannabis co-use is conceptualized and measured. Few studies examined simultaneous use specifically or disentangle co-use from broader polysubstance patterns. Research prioritizes standardized and event-level assessment can enhance accuracy of measurement and elucidate contextual factors for alcohol and cannabis co-use. Understanding how alcohol and cannabis co-use affects populations disproportionately impacted by HIV can inform more effective and tailored HIV treatment and prevention strategies.

## Introduction

Alcohol and cannabis are commonly used substances among adults in the United States [1]. Based on the 2024 National Survey on Drug Use and Health (NSDUH), 47.5% and 51.0% of adults aged 18 to 25 and aged 26 or older, respectively, reported past-month alcohol use; 24.1% and 15.1% of adults aged 18 to 25 and aged 26 or older, respectively, reported past-month cannabis use [2]. The rapidly growing cannabis legalization in the United States not only increases cannabis use but also increases concurrent (i.e., use of both substances, but not necessarily with overlapping effects) or simultaneous use (i.e., substance use effects overlap) of alcohol and cannabis [3, 4]. Since the first recreational cannabis legalization in Colorado and Washington in 2012, 24 states have legalized cannabis for recreational use [5]. Research has found that recreational cannabis legalization is positively associated with an increase in co-use of alcohol and cannabis among adults across all age groups and socioeconomic status [3, 4]. Specifically, 2019 NSDUH data show that 10.0%, 6.1%, and 3.6% of adults aged 21–30, 31–40, and 41–50 reported simultaneous use of alcohol and cannabis in the past month, respectively [1]. With the ongoing cannabis legalization changes, it is particularly important to understand the associations between alcohol and cannabis co-use (which includes concurrent use and simultaneous use) and its compound comorbidity with other health outcomes, such as HIV.

The prevalence of alcohol and cannabis use is especially high among people with HIV and people at risk for HIV [6–8]. Research has consistently shown that alcohol use, especially harmful levels of alcohol use, are strongly associated with negative HIV treatment and prevention outcomes such as lower HIV pre-exposure prophylaxis (PrEP) use [9–12], worse antiretroviral medications (ARVs) adherence [13–15], and greater engagement in sex behaviors that are associated with HIV transmission [11, 16]. However, results on the associations between cannabis use and HIV treatment and prevention outcomes have been limited and less consistent. While cannabis use has been associated with worse PrEP and ARV adherence [9, 17], more missed HIV care visits [18], and more condomless anal sex [19, 20], other studies have found no association between alcohol and cannabis co-use and PrEP [21, 22] or ARV adherence [20, 23]. Yet to date, the literature on alcohol and cannabis co-use has been conducted largely among college students or general community populations [24–27]. These studies have found that alcohol and cannabis co-use is associated with higher rates of negative consequences, such as levels of intoxication and physiological effects (e.g., feeling high, dizzy, confused) [25, 28], greater frequency and quantity use of each substance [25, 29], and greater risks of illicit drug use [30]. Whereas most HIV-related studies on alcohol and cannabis use focus on a single substance – either alcohol or cannabis use, given the critical role of alcohol and cannabis co-use in HIV treatment and prevention research – it is important to synthesize literature that addresses alcohol and cannabis co-use to understand how co-use may result in synergistic effects that may hinder engagement with HIV treatment and prevention.

Empirical evidence of the relationship between alcohol and cannabis co-use and HIV treatment and prevention requires a quantifiable measurement of co-use [25, 31]. A recent review on simultaneous alcohol and cannabis use among young adults aged 18 to 30 indicated the inconsistency of the findings regarding patterns of use and consequences of use, possibly due to wide variation in the definition and measurement being used across studies [32]. Despite growing attention to alcohol and cannabis co-use in HIV research, co-use is mostly used as a broad term, encompassing the use of alcohol and cannabis at the same time (i.e., simultaneous use, with overlapping effects) or in a close period, which may or may not include overlapping effects (e.g., effect chasing, use of each substance on different occasions or on different days). Differentiating co-use from simultaneous use is important, as simultaneous use may pose a particular risk: the overlapping effects of alcohol and cannabis can create synergistic harms that compound comorbidities and hinder HIV care engagement. While some studies have reviewed co-use or simultaneous use in terms of general health outcomes, to date, no reviews have considered these patterns in the context of HIV, in which substance use and HIV are a major syndemic condition faced by populations vulnerable to HIV. Further, the association between alcohol and cannabis co-use and HIV treatment and prevention outcomes is likely moderated by broader social-structural factors, such as HIV-related stress, unstable housing, and financial hardships, that shape both substance use patterns and HIV-related outcomes. Considering these contextual factors is critical for understanding the mechanism in which alcohol and cannabis co-use affects HIV treatment and prevention outcomes.

This study aims to summarize how alcohol and cannabis co-use is measured across studies in the HIV treatment and prevention literature. We also aim to qualitatively synthesize the existing evidence pertaining to the relationship of alcohol and cannabis co-use and HIV treatment and prevention, including HIV biomedical intervention (e.g., HIV PrEP and ARVs) and sex behaviors.

## Methods

### Protocol and Registration

We conducted a scoping review of the published literature to summarize results of studies examining alcohol and cannabis co-use and HIV related risk and care engagement outcomes. This review was based on a published protocol [33], and was developed using the Preferred Reporting Items for Systematic reviews and Meta-Analyses extension for Scoping Reviews (PRISMA-ScR) [34]. A risk of bias assessment was not conducted, in alignment with guidelines for scoping reviews [34].

### Eligibility Criteria

Included studies could be original, empirical research of any study design, including both qualitative or quantitative evidence types. Articles included were published in the English language and located in any global area. They could pertain to populations of any age group, sexual or gender orientation, race or ethnicity. We included only articles that detailed HIV treatment and prevention outcomes relating to HIV status and/or HIV transmission risk, including but not limited to PrEP or ARV use/adherence or HIV transmission potential based on sex behaviors. Additionally, studies must contain descriptions, conceptualizations, or measurements of polysubstance or co-use pertaining specifically to alcohol and cannabis. Articles were excluded if they did not mention HIV treatment and prevention outcomes or HIV risk, or if they mentioned patterns of drug use or co-use not relating to alcohol and cannabis.

### Search Methods

After harvesting key terms and seed articles, a comprehensive search strategy was designed by a research librarian trained in evidence synthesis methods (JM, a social sciences and public health librarian). The strategy included Boolean logic, advanced search punctuation (such as truncation), and both controlled vocabulary (such as MeSH terms) and natural language keywords. The strategy was translated into the databases and indexes listed below. Full search strategies for all databases can be found in the supplementary material (Supplemental Table 1). Databases searched included: PubMed, Ovid MEDLINE, Ovid PsycInfo, CINAHL (EBSCOHost), Web of Science (Arts & Humanities, Social Sciences, and Science Citation Indexes), Embase, and Scopus. Both peer-reviewed and grey literature, including conference abstracts, dissertations, and reports, were sought through the above databases. No date limit was applied to the search. The search was initially run in November 2023, and was re-run in December 2024 to harvest newly-indexed and newly-published studies.

### Screening of Studies

Search results from all databases were imported into Covidence software for screening. The screening process was piloted using 25 random title and abstract results [35], to ensure inter-rater reliability between screeners. Screening did not take place until 75% agreement or higher was reached between piloting screeners (MM and CM). As recommended by the Cochrane Handbook of Systematic Reviews of Interventions (Sect. 4.5) [36], screening included first a review of all title and abstracts, followed by a round of full-text evaluation. Two authors (MM and CM) screened each article, and a third author (YC) acted as a tiebreaker in the case of disagreement between the primary screeners.

**Fig. 1:**
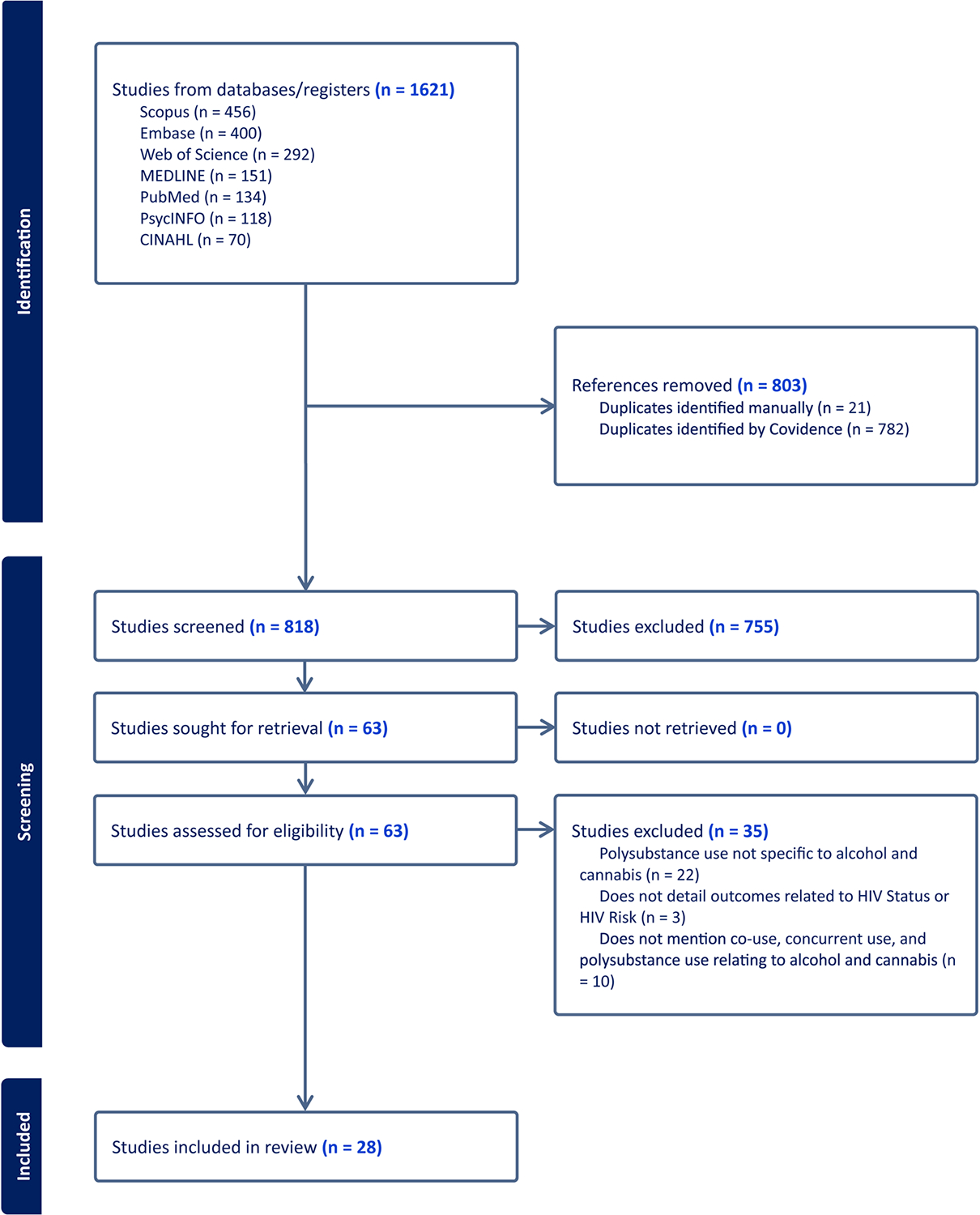
PRISMA flow chart outlining study search and review

1,621 studies were located after searching in all databases. Deduplication was conducted through Covidence [37, 38], and 818 studies remained for title/abstract screening. 63 articles were sought for full-text review, and 28 studies were identified for inclusion in the review. Of those articles not included in the analysis, reasons for exclusion included polysubstance use not specific to alcohol and cannabis (*n* = 22), did not include detail related to HIV status or HIV risk (*n* = 3), and did not mention co-use, concurrent/simultaneous use, and polysubstance use relating to alcohol and cannabis (*n* = 10). The full PRISMA flow chart of screening and included studies is included in Fig. 1.

### Data Charting and Synthesis

Data were extracted using Covidence software, using a custom extraction template developed by the authors. The extraction process was also piloted for reliability, with two authors (MM and CM) using the charting template to extract data from five articles and compare them until a consensus was reached. Extraction was performed by two authors (MM and CM), with a third (YC) checking for consensus. As this article is a scoping review, no meta-analysis or critical appraisal was performed.

## Results

### Sample and Study Characteristics

Studies (*n* = 28) were predominantly conducted with sample populations within the United States (*n* = 20), with three studies from Canada, two studies with a sample in South Africa, one study from the Netherlands, one from Mexico, and one that included a sample from both Brazil and Thailand. Overall, ten of the studies defined study populations inclusive of sexual and gender minorities; eleven studies included a study population comprised of people with HIV. Age was not considered in either the inclusion or exclusion criteria; thus, studies could include adults and adolescents. Below, we summarize the key characteristics and findings of each study; note that a study may be discussed in multiple sections if the study examined multiple health outcomes.

### Measurement and Operationalization of Alcohol and Cannabis co-use

Of the 28 included studies, 17 did not specifically measure alcohol and cannabis co-use; instead, they explored alcohol and cannabis co-use in the context of polysubstance use through cluster analyses, such as latent class analysis (LCA). Of these 17 studies, all studies interpreted at least one latent class profile as including membership with high probabilities of using both alcohol and cannabis. Notably, most studies included latent class factors indicating drug use beyond alcohol and cannabis (i.e., stimulants, methamphetamine, poppers, etc.), with one study including sociodemographic characteristics as well as substance use variables to construct latent classes.

Two studies did not explicitly create a polysubstance use measure and instead assessed alcohol and cannabis co-use through an interaction term of both alcohol and cannabis in the statistical models [39, 40].

Five studies assessed alcohol and cannabis co-use by creating a composite variable based on individually reported substance use; however, the time frame, frequency, and intensity of use for both or either substance use varied substantially by study. Two studies incorporated a daily measure [41, 42]. One study defined concurrent use via frequency of cannabis use (> 10 days/month) and unhealthy alcohol use (AUDIT score ≥ 8) [41]; the other study defined concurrent use as alcohol and cannabis use on the same day [42]. Similarly, one study measured alcohol and cannabis co-use through both self-report (AUDIT score ≥ 8) and confirmation of drug use in the last 48 h with a urine drug screen [43]. Two studies defined alcohol and cannabis co-use by reporting alcohol and cannabis use during the past month [44, 45].

Four studies created a composite variable of polysubstance use; these measures included alcohol and cannabis use, but did not create a standalone category of alcohol and cannabis co-use [46–49]. One study specified current use of alcohol and cannabis, but did not provide a time frame [47]; another measured substance use in the last three months [49]. Lastly, one study measured substance use during sex in the past six months [48].

### HIV Treatment and Prevention Outcomes

#### Clinical HIV outcomes (CD4 cell count, viral suppression, viral load)

Five studies examined biomedical HIV treatment and prevention outcomes among people with HIV (PWH), such as CD4 cell count, viral suppression, and viral load [46, 49–52]. In two studies that used LCA to summarize polysubstance use patterns among PWH, latent profiles including predominantly alcohol and cannabis use were associated with higher HIV viremia or lower CD4 cell counts in comparison to lower substance use latent profiles, but not in fully adjusted models [50, 52]. Similarly, one study, including people from Brazil and Thailand, found no association between the number of substances used and decreased viral load; though, it should be noted that this study did not include a standalone measure of alcohol and cannabis co-use [49]. Further, data from a longitudinal study with 5,023 PWH in Washington, DC suggests that people in the Polysubstance Use latent class (substance use profile: 79% alcohol, 42% cannabis, 94% stimulant) were more likely to be virally unsuppressed over time compared to people in the Low-Level Substance Use class (substance use profile: 24% alcohol, 8% cannabis, 4% stimulant).

#### ARV adherence

Six studies found a relationship between polysubstance use involving alcohol and cannabis and suboptimal ARV adherence [43, 46, 47, 49, 53, 54]. For example, a cross-sectional study conducted among 391 PWH found that participants with both harmful alcohol and cannabis use missed more doses of their ARVs compared to participants who did not use any substances [43]. Another study found women with suboptimal ARV adherence were more likely to be classified in the Socially Acceptable Polysubstance Use class (characterized by high probabilities of alcohol, cannabis, and tobacco use) compared to women with optimal ARV adherence [54]. Three studies employed composite measures of polysubstance use; all three studies identified an association between polysubstance use and lower ARV adherence [46, 47, 49], with two studies noting that a higher number of substances used was associated with lower adherence [46, 49]. These studies used composite measures of polysubstance use, which included alcohol and cannabis, but did not include a standalone category of only alcohol and cannabis.

#### Pre-exposure prophylaxis (PrEP) and post-exposure prophylaxis (PEP)

Few studies examined the relationship between alcohol and cannabis co-use and pre-exposure prophylaxis (PrEP). Only one study examined knowledge and perceptions of PrEP, postexposure prophylaxis (PEP), and the benefits of HIV treatment outcomes among sexual and gender minority men [55]. The authors identified six latent profiles; at least two of the latent profiles involved high frequencies of alcohol and cannabis use (i.e., Common Drug and Club Drug classes). The Common Drug class was marked using alcohol, tobacco, cannabis, and poppers: 96.5% drank alcohol in the past year, 43.3% smoked, 73.9% used cannabis, and 37.8% used poppers. The Club Drug class had higher alcohol (9.8% reported daily use, 56.4% reporting drinking alcohol at least a few times a week), cannabis (90.1%), cocaine (76.3%), ecstasy (63.0%), and stimulant (16.6%) use. The study found that those classified in the Common Drug and Club Drug classes were more likely to have knowledge concerning ARV, PrEP, PEP, and have interest in PEP use, in comparison to men in the Limited Drug Use class (marked by the limited use of substances). No associations were found among men with HIV. One other study found that men not living with HIV who were on PrEP were more likely to report sex events characterized by Common Drug use (marked by cannabis, alcohol, and popper use) in comparison to men not living with HIV and who were not on PrEP [56].

#### HIV testing and diagnoses

Two studies measured HIV testing and diagnoses [57–59]. Repeat HIV testing was less likely among people classified as High Polysubstance Use (characterized by high probabilities of use of stimulants, cannabis, and hallucinogens), compared to a Low Substance Use class; however, this association was not significant when comparing the High Cannabis, Stimulant, and Alcohol Use class to the Low Substance Use class [57]. Mild polydrug use with severe alcohol and cannabis use was associated with HIV diagnoses among men and women [58].

### Sexually Transmitted Infections (STIs)

Six studies examined the associations between alcohol and cannabis use and STI prevalence [41, 60–64]. Among these six studies, two explicitly operationalized STI prevalence as inclusive of HIV [60, 61], two did not include HIV as part of their definition of STIs [62, 63], and two studies did not specify which STIs were included [41, 64]. Several studies found a relationship between alcohol and cannabis co-use and STI prevalence [41, 61, 64]. High concurrent use of both alcohol and cannabis was associated with increased likelihood of STI diagnoses [41]. One study which operationalized alcohol and cannabis co-use via longitudinal latent profile analysis found that the class characterized by high use of both alcohol and marijuana had the highest proportion of STI diagnoses; the second highest proportion of STI diagnoses was among the group characterized by moderate alcohol and increasing cannabis use [64]. (Table 1)

However, some studies suggest a need to examine in greater detail the specific combinations of substances and their impact on STI prevalence. In one study, the Polydrug Use class (characterized by high probability of amphetamine, GHB/GBL, ketamine, methamphetamine, mephedrone and XTC use) was associated with higher odds of STI prevalence compared to the No Drug use class [60]. However, the Few, Various Drugs class (characterized by high probabilities of alcohol, cannabis, cocaine, GHB/GBL, nitrites and XTC use) and the Alcohol and Soft Drugs (marked by high probabilities of alcohol, cannabis, nitrites and XTC) did not have a significantly higher STI prevalence when compared to the No Drugs class [60]. A similar pattern was observed in another LCA-based study [63]. Lastly, another study which operationalized alcohol and cannabis co-use via LCA found no difference between LCA clusters and STI prevalence [62].

One study constructed LCA profiles based on substance use and STI-related measures [61]. The authors found that women categorized in a latent class characterized by frequent cannabis use and occasional alcohol use were more likely to report an STI in the past year, in comparison to women reporting low alcohol and cannabis use; however, this difference did not persist beyond ages 18–19 [61].

### Sex Behaviors that Could Increase Risk of HIV

Overall, 13 studies assessed the relationship between alcohol and cannabis co-use and sex behaviors such as condomless anal sex and the number of sex partners [39–42, 45, 48, 56, 58, 62, 64–67]. Multiple studies found an association between substance co-use and engagement in sex behaviors that could lead to increased HIV transmission. Descriptively, one study examined perceived HIV risk among women using multiple substances, finding that all women (*n* = 3) who reported alcohol and cannabis use perceived a 25% risk of acquiring HIV [44]. Concurrent or polysubstance use involving alcohol and cannabis was largely associated with higher odds of having multiple sex partners, inconsistent condom use or lower condom intentions, engagement in or pressure to engage in condomless anal sex, and an age gap with a partner of more than 5 years in the last 3 months compared to people reporting no substance use [41, 42, 45, 48]. The effect estimates were higher for participants reporting alcohol and cannabis co-use along with another drug [45]. These findings are reflected in LCA-based studies [56, 64, 67]. For example, one study, which used LCA to characterize patterns of alcohol and cannabis co-use, found that the group marked by moderate alcohol use and increasing cannabis use had a higher frequency of condomless sex and a higher proportion of multiple sex partners when compared to people who did not use either substance [64]. Similarly, another study found that sex behaviors which could increase HIV transmission (e.g., higher number of sex partners, condomless anal sex) were more likely in the class characterized by high probabilities of alcohol, cannabis, and tobacco use compared to the class characterized by low levels of any drug use [56].

However, some evidence suggests that this relationship could differ by gender. Houck et al. found no difference between relevant LCA clusters and sex behaviors which could increase HIV transmission among men; however, women in the class characterized by alcohol and cannabis use (and condomless sex) reported a higher number of partners compared to the lowest risk group and were more likely to engage sexually with partners with higher HIV/STI transmission behaviors [62]. Similarly, women categorized in the class characterized by polydrug use were more likely to have a higher number of sex partners engaging in condomless sex in comparison to women in classes characterized by [1] mild polydrug use with severe alcohol and cannabis use, and [2] alcohol and cannabis use [58]. This relationship was not observed among men.

Similar to findings related to STI prevalence, there is evidence to suggest that the types of drugs people use influence sex behaviors that could increase HIV transmission. An LCA-based study found that women in the class characterized by alcohol and cannabis use had higher odds of vaginal intercourse (considered less likely to transmit HIV than anal sex) compared to women in the group characterized by no substance use; however, the group characterized by drug use beyond alcohol and cannabis was less likely to report vaginal intercourse and more likely to report anal intercourse [65]. Another study found that women categorized in a class characterized by alcohol and cannabis use had lower odds of reporting condomless sex in the last month in comparison to women in a class characterized by polysubstance use (inclusive of high probabilities of methamphetamine and cannabis use, and heavy drinking) [66].

Lastly, one study operationalizing co-use with an interaction term found that alcohol and cannabis co-use does not impact sex behaviors [39]. However, another study which also operationalizes alcohol and cannabis co-use through an interaction term found the simultaneous use of these substances was associated with increased risk of sex behaviors that could increase HIV susceptibility [40].

## Discussion

This scoping review sought to qualitatively examine the relationship between alcohol and cannabis co-use and HIV treatment and prevention outcomes. Our review did not restrict any sub-populations and included studies that were conducted among populations of any age group, sexual or gender orientation, or race or ethnicity. We found that the literature examining the association between alcohol and cannabis co-use and HIV treatment and prevention outcomes is sparse relative to that assessing cannabis and alcohol separately, with heterogeneity in measurement of co-use and methods of analysis employed. Of note, we did not identify any qualitative studies regarding alcohol and cannabis co-use and HIV treatment and prevention outcomes.

The conceptualization and operationalization of alcohol and cannabis co-use varied substantially across the identified articles, with heterogeneity in the reporting time-frames for co-use (e.g., past month, same day, no time frame reported). Most of the included studies did not specifically define alcohol and cannabis co-use as simultaneous use, concurrent use, or sequential use. Among the 28 articles included, only one study explicitly measured simultaneous use by using a daily survey and timeline follow back to assess alcohol and cannabis use [42]. The oblique definition of co-use across this review reflects a longstanding and previously recognized limitation of much of the literature assessing the potential impacts of polysubstance use [31]. Prior reviews have identified that those using alcohol and cannabis simultaneously may experience more negative consequences compared to those using alcohol or cannabis alone; however, findings were more mixed when comparing simultaneous use to concurrent use [32]. Differentiating simultaneous use, where the effects of the substances overlap, from concurrent use is an important consideration when examining typologies of co-use and HIV treatment and prevention outcomes. Furthermore, patterns of co-use that also consider factors such as the sequence of use, frequency, quantity of co-use and social context of use, are also critically important for understanding the mechanism of the effects of co-use on HIV treatment and prevention outcomes [68].

In addition to the inconsistency in the reporting time-frames, there is heterogeneity in analytic approaches to examining alcohol and cannabis co-use, particularly in the context of polysubstance use. Many studies included in this review utilized cluster analyses, such as LCA [50–53, 55, 56, 60, 63–67]. Studies that utilized an LCA methodology identify different subgroups of individuals based on distinct substance use profiles, including alcohol and cannabis use. However, LCA methodologies often preclude an examination on the main effect of alcohol and cannabis co-use (e.g., a class with relatively higher probabilities of alcohol and cannabis use, these classes often included probabilities of other substance use or the comparator was a class that also included alcohol and cannabis use). For example, in Byrne et al. participants in a class termed Polysubstance Use were more likely to be virally unsuppressed over time compared to those in the Low-Level Substance Use class, yet while there were comparatively high levels of cannabis and alcohol use in the Polysubstance use class, such that stimulant use was the most prevalent substance in the profile [51]. Evidence shows that alcohol and cannabis are commonly present in the context where individuals use multiple substances. A robust conceptualization of alcohol and cannabis co-use in the context of polysubstance use is underdeveloped. Future research on alcohol and cannabis co-use among people at-risk for or with HIV needs to differentiate alcohol and cannabis co-use as a discrete combination from other polysubstance use typologies [31].

As of the time we conducted this review, we only identified two studies that examined alcohol and cannabis co-use and PrEP-related outcomes. For example, in Card et al. (2023), men who were on PrEP were more likely to report Common Drug Use (i.e., combined use of alcohol, cannabis, and poppers) during sex relative to men who were not on PrEP [56]. Similarly, in Card et al. (2020), for men not living with HIV or men with unknown HIV serostatuses, those in the Common Drug class had more HIV and PrEP knowledge than those in the Limited Substance Use class [55]. Such findings suggest the possibility that, depending on the context of use, alcohol and cannabis co-use may not be an assumed barrier to PrEP care continuum outcomes. While several past studies have found the detrimental effect of harmful alcohol use on PrEP use and adherence [10, 12, 69], a 2021 scoping review suggested conflicting results in the relationship between alcohol use and PrEP awareness and adherence [70]. Similarly, limited literature also found an inconsistent association between cannabis use and PrEP outcomes [9, 22, 71], It is clear that there is a dearth of research on alcohol and cannabis co-use and PrEP outcomes, and additional studies that assess situational and event-level associations are necessary to further investigate this relationship. (Table 2)

In terms of ARV-related outcomes (e.g., ARV adherence, viral suppression, CD4 counts), it appears that alcohol and cannabis co-use is related to worse ARV related outcomes, such as medication adherence and viral suppression, among PWH. For example, in Degarege et al., the mean number of missed ARV doses over the past four days was greater among participants who used alcohol and marijuana compared to those who reported no substance use [43]. Two studies assessed viral load, finding that polysubstance use classes with comparatively higher probabilities of alcohol and cannabis use to other classes were positively associated with unsuppressed HIV [51, 52], though this relationship was no longer significant in one study in multivariable models [52]. It should be noted that none of these studies explicitly examined alcohol and cannabis co-use as a discrete combination. Instead of evaluating the specific role of alcohol and cannabis co-use, all these studies assessed alcohol and cannabis in the context of polysubstance use. It is unknown if the observed associations between alcohol and cannabis co-use and ARV outcomes are specifically attributed to alcohol and cannabis.

Many of the studies found significant relationships between alcohol and cannabis co-use and sex behaviors that could increase HIV transmission. These findings align with extant literature identifying a relationship between alcohol use and cannabis use separately on sex behaviors that may increase HIV transmission [20, 72]. Notably, similar to the issue identified earlier, such findings should be interpreted with caution in the context of polysubstance use, whereas other substance use may serve as a confounder. For example, in a study by Wechsberg et al., women in the Marijuana and Alcohol class had lower odds of reporting condomless sex in the past month with their main sex partner compared with women in the Polydrug Use class [66]. However, while some of these studies also included other substance use in their analyses (e.g., in the latent class profile) or did not control for other substance use, cannabis and alcohol co-use alone may still have a distinct impact. In the study by Arrington-Sanders et al. while the effect estimates for alcohol, cannabis and other substance use were larger than those for alcohol and cannabis co-use only, those in the alcohol and cannabis co-use class were still more likely to engage in sexual risk behaviors, including inconsistent condom use, than those reporting no substance use [45].

## Limitations

The findings of this review should be interpreted alongside limitations. Although we developed comprehensive search strategies with the help of a social sciences and public health librarian, it is possible that some articles were not included. One of our inclusion criteria was the centrality of co-use of alcohol and cannabis. We conducted a fully transparent review in which two highly trained researchers conducted screening independently and included search terms that we believe best represent co-use. However, given the heterogeneity of the definition of co-use, other researchers may have chosen articles that we excluded. This review did not restrict the study populations and the definitions of alcohol and cannabis co-use. Therefore, there is a wide variation in terms of the results that preclude us from performing any pooled analyses to evaluate the strength of the evidence. Overall, we identified few studies that explicitly measured alcohol and cannabis co-use; future work should more carefully examine the effects of alcohol and cannabis co-use, with particular emphasis placed on precise measurements of simultaneous or concurrent use and ensuing effects on HIV treatment and risk. The lack of literature in this area is compounded by varying definitions of co-use, which must be made more consistent across the literature in order to facilitate comparisons between studies. As additional studies are generated, evidence can then be compared across geographic contexts, which is a limitation of this study; given the small number of studies identified, with the vast majority originating in the United States, a comparison between geographic areas was not feasible. Future studies should be conducted globally to understand how the legislative environment and various cultural contexts impact alcohol and cannabis co-use and its relationship with HIV treatment and prevention. No qualitative studies were identified based on our review protocol. Future qualitative studies are needed to provide insights into the associations between alcohol and cannabis co-use and HIV-related outcomes, to examine potential moderators and mediators, and to identify mechanisms that could inform strategies to promote HIV treatment and prevention and reduce harms associated with alcohol and cannabis co-use. Lastly, given the small number of studies identified, and in alignment with our protocol, we included studies that specifically mentioned alcohol and cannabis use, but did not necessarily include a standalone category of alcohol and cannabis use (*n* = 4). While this may impact comparisons, we note where these specific studies are referenced and not in alignment with findings from other studies.

## Conclusions

This review is the first scoping review to examine the associations of alcohol and cannabis co-use and HIV treatment and prevention outcomes. With the prevalence of alcohol and cannabis co-use continuing to increase under a rapidly evolving cannabis legalization environment, much work remains to be done to address this gap in the literature to better understand the role of alcohol and cannabis co-use in HIV treatment and prevention. Specifically, studies that utilize detailed measures of co-use, including differentiating between simultaneous and concurrent use and patterns of co-use, are needed to ascertain nuances of alcohol and cannabis co-use typologies and their relationship to HIV treatment and prevention outcomes. Event-level or situational-based data collection is recommended to collect momentary data that reflect the discrete combinations of alcohol and cannabis use in relation to HIV/STI prevention and transmission behaviors and health outcomes. Future research should also examine the role of alcohol and cannabis in the context where multiple other substances (e.g., nicotine, stimulants) are involved.

## Supplementary Material

Supplementary tables

**Supplementary Information** The online version contains supplementary material available at https://doi.org/10.1007/s40429-025-00707-x.

## Figures and Tables

**Table 1:** Summary of all the studies included in this scoping review

Authors	Year	Aim	Population	Study Design	Sample Size	Study years	Exposure or predictors	Outcome
Achterbergh et al. [60]	2020	Examine differences in sexualized drug use among MSM in Amsterdam and surrounding urban regions, assess patterns in sexualized drug use, as determined by identifying latent classes of drug use, and assess their association with risk behavior and STI prevalence	Netherlands: Men who have sex with men in Amsterdam and surrounding urban regions	Cross-sectional	4,461	2017	LCA profiles based on drug use	Sex behavior and other STI risk factors in the preceding six months
Arrington-Sanders et al. [45]	2024	Characterize patterns of polysubstance use and explore associations between substance use and HIV risk behaviors	USA: Young Black and Latinx SMM and TW living in four cities in the US, (Baltimore, Washington, Philadelphia, and Tampa/St. Petersburg)	Cross-sectional	466	2017–2021	Polysubstance use	Sex behaviors, including inconsistent condom use and having an older male partner in the last 3 months, ever experiencing pressured sex to engage in sex without a condom
Bertholet et al. [50]	2023	Identify polysubstance use patterns in a cohort of people with HIV with alcohol and/or other SUDs, and assess associations with HIV disease severity	USA: Adults living with HIV in the Boston area	Longitudinal cohort	250	Enrollment between 2012–2014, follow-up 12 months later	LCA profiles based on substance use	CD4 cell count, HIV viral suppression (HIV viral load < 200 copies/ml)
Brown et al. [44]	2006	Describe perceptions of risk accompanying risk behavior patterns among rural African American women who use substances	USA: African American women who use drugs, in a rural north Florida community	Mixed methods (cross-sectional survey)	30	Not reported	Substance use	HIV risk behavior
Byrne et al. [51]	2024	Determine the association between substance use patterns and HIV RNA trajectories	USA: People with HIV in Washington, DC	Longitudinal cohort	5,023	2011–2019	LCA profiles based on substance use	Viral load trajectories
Card et al. [56]	2023	Use event-level data to describe patterns in sexualized drug use among gbMSM, to identify person-level and event-level factors associated with patterns of use	Canada: Gay and bisexual men who have sex with men in Metro Vancouver	Longitudinal cohort	774	2012–2019	Correlates of LCA membership including HIV-related measures	LCA profiles based on event-level substance use
Card et al. [55]	2020	Examine the associations between patterns of substance use among gbMSM and their awareness of the preventive benefits of ART, PrEP, and post-exposure prophylaxis (PEP)	Canada: Gay and bisexual men who have sex with men in Canada	Cross-sectional	7,991	Oct 2014 - April 2015	LCA profiles based on substance use	Awareness and knowledge of PrEP, PEP, and preventive benefits of HIV treatments
Carney et al. [57]	2023	Identify substance use typologies among a sample of SGMY who use substances and examine whether these typologies are associated with sexual health outcomes	USA: Sexual and gender minority men who engage in sex with men in Detroit, Michigan	Cross-sectional	414	Apr 2017 - Sept 2019	Sexual health variables (HIV testing, diagnosis of STI, PrEP awareness and use)	LCA profiles based on alcohol and drug use variables
Carter at al. [54]	2018	Examine associations between multiple types of substance use with cART adherence, interpersonal violence, and other social determinants of health	Canada: women living with HIV	Cross-sectional	1,424	Aug 2013 - May 2015	ART nonadherence, never/not currently on cART	LCA profiles based on 18 survey questions related to substance use
Choi et al. [41]	2022	Characterize the adverse sexual health, mental health, and social factors associated with frequent marijuana use and unhealthy alcohol use among young Black women	USA: Young Black women ages 17–24 in Atlanta	Cross-sectional	560	Not reported	Frequent concurrent use of marijuana and alcohol	Biologically-confirmed STI, multiple sex partners, recent transactional sex, lower condom intentions
Chung et al. [61]	2022	Identify profiles of young women’s perceived partner alcohol and cannabis use, sex behaviors, and STI risk	USA: Community-based sample of girls and women in Pittsburgh from ages 18–20	Longitudinal cohort	2,450	Not reported	NA: Descriptive analysis of sex behaviors and STI diagnosis among latent class analysis (LCA) profiles	
D’Anna et al. [39]	2021	Explore the role of problem cannabis use to the sexual risk profile of YBMSM	USA: Young black sexually minoritized men in California	Cross-sectional	250	Nov 2016 - June 2018	Alcohol, cannabis use	Sexual health risk index, HIV/STI risk
Degarege et al. [43]	2022	Examine the relationship of polysubstance use with ART adherence and evaluated whether depression moderated this relationship	USA: Minority adults residents in Miami-Dade county	Cross-sectional	391	2014–2016	Polysubstance use (including alcohol and cannabis)	ART adherence
El-Bassel et al. [58]	2019	Examine different types of drug use typologies among men in community correction programs and their female primary sex partners	USA: Men in community correction programs in New York City	Cross-sectional	1,209	Not reported	Sex behaviors	LCA profiles based on drug use
Firkey et al. [42]	2021	Examine the event-level association between cannabis and alcohol co-use and CAS with a partner of any HIV status	USA: HIV-positive men who have sex with men in Syracuse, NY and San Francisco, CA	6-week timeline followback interview of recent substance use and sexual activity plus another 6-week of telephone interview	101	Not reported	Day-level substance use	Condomless anal sex with a partner of any HIV status and with a partner of negative/unknown HIV status
Green et al. [64]	2017	Identify the relationship between patterns of alcohol and marijuana use during adolescence and sexual risk behaviors and outcomes in young adulthood	USA: Black youth living in Baltimore city	Longitudinal cohort	678	1993 (1st grade) - Age 25	LCA profiles based on substance use	Sexual outcomes: multiple sexual partners, sex without a condom (past month), teenage pregnancy, and contraction of an STI
Houck et al. [62]	2006	Inform HIV/STI prevention among adolescents by examining demographic and behavioral risk factors associated with HIV	USA: Youth (15–21) engaged in an HIV prevention program in three US cities (Atlanta, Providence, Miami)	Cross-sectional	1,412	Not reported	NA: Cluster analysis with three clusters: (1) mental health crises/unprotected sex, (2) alcohol and marijuana use/unprotected sex, and (3) lower risk	
Ma et al. [46]	2025	Examine the associations between substance use severity (including number of substances used) with ART adherence	USA: People with HIV in the Centers for AIDS Research Integrated Clinical Systems, includes eight academic sites	Longitudinal cohort	10,557	2010–2021	Alcohol and drug use severity, including polysubstance use	ART adherence in the last 30 days
Przybyla et al. [47]	2022	Assess the relationship between polysubstance use and ART adherence	USA: People with HIV in the Bronx, NY	Cross-sectional	298	Recruited May 2017–April 2018	Polysubstance use	ART adherence (any reported missed doses of ART in the last seven day)
Reynolds et al. [65]	2019	Understand drug use patterns before or during sex to understand risk reduction practices	USA: Women in Long Beach, California, in a low socioeconomic area	Cross-sectional	812	Not reported	LCA profiles based on substance use	Sex behaviors (past-30 days for vaginal or anal intercourse, oral sex, HIV/STI positivity)
Ritchwood et al. [40]	2016	Determine whether specific drugs differently relate to sex behaviors	USA: Black impoverished youth in Mobile, Alabama	Longitudinal cohort	9,477	1998–2008	Alcohol, cannabis use	Sex behaviors
Rodriguez-Bolanos et al. [67]	2022	Identify subgroups with specific substance use patterns and associations with HIV risk behaviors among MSM	Mexico: Sexual minority men and transgender women in Mexico City	Cross-sectional	2,027	May 2018–Dec 2019	LCA profiles based on substance use	Sex behaviors, including condomless anal sex in the past 3 months, or serosorting
Rosen et al. [52]	2024	Identify patterns of substance use and examine their relationship to HIV viremia	South Africa: Female sex workers living with HIV in Durban	Cross-sectional	1,391	Enrollment from Jun 2018-Mar 2020	LCA profiles based on substance use	HIV viremia
Shrader et al. [48]	2024	Examine the sexual network-level associations of condom use during anal sex.	USA: Black SMM and TW in Chicago, Illinois	Cross-sectional	412	Jan 2018–Dec 2019	Polysubstance use during sex	Condomless anal sex
Tobin et al. [63]	2016	Define and characterize drug and alcohol use patterns among African American MSM and examine differences in sexual risk by pattern of use	USA: Black MSM in Baltimore	Cross-sectional (two samples)	369	Aug 2007–Aug 2008; Mar 2012–Jul 2012	LCA profiles based on substance use	Sex risk and HIV status
Tsuyuki et al. [49]	2019	Examine the overlap between substance use, depressive symptoms, ART adherence, and HIV viral load undetectability	Brazil, Thailand: men living with HIV in Rio de Janeiro (Brazil) and Chiang Mai (Thailand)	Longitudinal cohort	305	Not reported	Polysubstance use	Viral suppression, ART adherence (in Thailand) and ART use (in Brazil)
Wechsberg et al. [66]	2012	Identify alcohol and other drug use typologies among women who use alcohol and other drug via LCA, and examine whether alcohol and other drug use classes predict sex risk for HIV	South Africa: Women ages 18–33 with HIV in Cape Town	Cross-sectional	720	Sept 2008 - Jan 2011	LCA profiles based on alcohol use, cannabis or other drug use	Unprotected sex with main sex partner, unprotected sex with sex partners other than main partner, alcohol or drug-impairment during last sex act
Wiginton et al. [53]	2024	Identify patterns of psychosocial conditions and assess how these are associated with HIV care outcomes among YLWH.	USA: Youth living with HIV in Atlanta, Chicago, Houston, New York, Philadelphia, Tampa	Cross-sectional	208	Enrollment from Aug 2019-May 2022	LCA profiles based on substance use and sociodemographic indicators	HIV care engagement, HIV treatment adherence, HIV viral load

**Table 2:** Summary of detailed descriptions of substance use and HIV treatment and prevention measures extracted from studies in this scoping review

Authors	Year	Exposure/predictors measure	Co-use measure	Outcome measure	Methods	Findings
Achterbergh et al. [60]	2020	LCA profiles based on: Drug use during sex in the past six months, including: *alcohol*, amphetamine, *cannabis*, cocaine, EDD, GHB/GBL, ketamine, methyl amphetamine, mephedrone, nitrites and XTC/MDMA	Did not explicitly measure co-use, used LCA. *LCA profiles:* Amsterdam sample: No drugs Alcohol Few, various drugs Polydrug Surrounding area: No drugs Alcohol and soft drugs Polydrug	Sex behavior and other STI risk in the past six months, STI diagnoses	LCA, run separately for samples in Amsterdam and surrounding regions, was used to distinguish classes of drug use during sex. Logistic regression to compare STI prevalence between clusters, with “no drug” as the reference group.	In Amsterdam, Polydrug Use had higher odds of STI prevalence compared to No Drug Use (aOR: 1.5; 95% CI: 1.1–1.9). The STI prevalence among Few, Various Drugs was not different than No Drugs. In the surrounding urban regions, Polydrug Use class had higher odds of STI prevalence (aOR: 1.9; 95% CI: 1.3–2.7) compared to the No Drug Use class. Alcohol and Soft Drugs class did not significantly differ from No Drug Use in terms of STI prevalence.
Arrington-Sanders et al. [45]	2024	*Alcohol use:* NIDA-Modified (NM) ASSIST to assess lifetime and recent (past 3 months) alcohol use *Cannabis use:* NIDA-Modified (NM) ASSIST to assess lifetime and recent (past 3 months) cannabis use	Composite variable using data from NM-ASSIST, including: (1) alcohol and cannabis, (2) alcohol, cannabis, and at least one other illicit drug, (3) alcohol or cannabis with one other illicit drug, (4) no lifetime use. Created based on most frequently used substances.	Sex behaviors, including inconsistent condom use and having an older male partner in the last 3 months, ever experiencing pressured sex to engage in sex without a condom	Multivariate logistic models accounting for confounders and geographic clustering	People who reported alcohol and cannabis had higher odds of inconsistent condom use (OR: 1.99; 95% CI: 1.45, 2.73), pressure to engage in condomless anal sex (OR: 2.67; 95% CI: 1.83, 3.90), and age gap with a partner of more than 5 years in the last 3 months (OR: 2.60; 95% CI: 2.05–3.30) compared to people reporting no substance use. Effect estimates were larger for participants describing alcohol, cannabis, and one other drug.
Bertholet et al. [50]	2023	LCA profiles based on substance use, including: *Alcohol:* Unhealthy alcohol use (AUDIT-C) *Cannabis:* Past 30-day use if cannabis (assessed using the Addiction Severity Index)	Did not explicitly measure co-use, used LCA. *LCA profiles:* None CA: mostly cannabis and alcohol use Multi: mostly opioids, cocaine, tranquilizers, cannabis, and unhealthy alcohol use	Past-year measure of CD4 cell count, HIV viral suppression (HIV viral load < 200 copies/ml)	Assessed the association between changes in patterns of use from baseline to 12 months and HIV disease progression using linear and logistic regression models.	Participants remaining in CA class had a significantly lower CD4 count compared with those with a favorable course in the model adjusted for baseline covariates, but not in the model adjusted for baseline covariates and baseline CD4 count. None of the associations were statistically significant in models looking at the absence of HIV viral suppression.
Brown et al. [44]	2006	*Alcohol*: Use in past 30 days (NIDA Social Network Questionnaire) *Cannabis*: Same as alcohol.	Combination of reporting alcohol and cannabis from the alcohol and cannabis use survey questions	*HIV risk behavior:* Use condom < 100% of the time (past 6 months), exchange sex for money, exchange sex for drugs, perceived risk of HIV	Descriptive statistics	Among the 22 women reporting use of the three drugs (alcohol, marijuana, and cocaine), 9 (41%) reported that they perceived no risk of acquiring HIV, 10 (45%) reported a 25% risk, and 3 (14%) reported at least a 50% chance. Among the 3 women reporting use of only alcohol and marijuana, 3 (100%) indicated that they had a 25% risk of acquiring HIV.
Byrne et al. [51]	2024	LCA profiles based on substance use indicators extracted from manual EHR chart abstraction and ICD 9/10 codes for substance use and dependence, including: *alcohol, cannabis*, stimulant, inhalant, hallucinogen, opioid, and sedative, hypnotic or anxiolytic use	Did not explicitly measure co-use, used LCA. *LCA profiles:* Low-Level Substance Use Opioid Use Polysubstance Use (highest probabilities of any group for alcohol and cannabis use, high probability of stimulant use)	Viral load trajectories (mean HIV RNA for each person during three, six-month periods)	Adjusted multinomial logistic regression models to identify an association between the LCA classes and HIV RNA trajectory outcomes.	The Polysubstance Use (aOR: 1.47; 95% CI: 1.07, 2.01) class was more likely to have unsuppressed HIV RNA trajectories over 18 months compared to the Low-Level Substance Use class.
Card et al. [56]	2023	*HIV risk:* HIV status, PrEP use in the past 6 months Also examine other potential correlates of LCA membership.	Did not explicitly measure co-use, used LCA. *LCA profiles:* Limited Use: limited use of any drugs Common Drug Use: alcohol, cannabis, poppers most used Licit Drug Use: use of alcohol alone Party ‘N’ Play Multi-use Cannabis+: cannabis, erectile drugs, and ecstasy	LCA profiles based on event-level substance use, including: *Alcohol*: Alcohol use 2 h prior to sexual event *Cannabis*: Cannabis use 2 h prior to sexual event	Multinomial logistic regression to identify factors associated with latent class membership.	Sexual events reported by participants living with HIV (vs. HIV-negative men not on PrEP) had higher odds of being classifed as Common Drug (aOR: 1.84; 95% CI: 1.72, 1.97), compared to the Limited Use class. HIV-negative men on PrEP were more likely to report events classified as Common Drug Use (aOR: 1.80; 95% CI: 1.41, 2.31) relative to HIV-negative men who were not on PrEP. Participants with more sexual partners in the past 6 months had higher odds of reporting Common Drug Use, compared to the Limited Use class. Condomless anal sex events were more likely among people classified in the Common Drug Class.
Card et al. [55]	2020	LCA profiles based on substance use, including: *Alcohol:* Frequency of use (any use, past 12 months, past month, past week, daily) *Cannabis:*Frequency of use in the past 12 months (any use, past 12 months, past month, past week, daily)	Did not explicitly measure co-use, used LCA. *LCA profiles* : Limited Drug Use (83% alcohol use, 6% cannabis use) Common Drugs: alcohol (96% past year use), cannabis (74%), tobacco (43%), poppers (38%) Club Drugs (56% past week alcohol use; 90% past year cannabis use) Sex Drugs (did not report % for alcohol or cannabis use) Prescription Drugs (did not report % for alcohol or cannabis use) Polydrugs (did not report % for alcohol or cannabis use)	Awareness and knowledge of PrEP, PEP, and preventive benefits of HIV treatments	Multivariable binary logistic regression models were created for each outcome (PrEP, PEP, and treatment benefits awareness, interest in PrEP [for HIV-negative men], and viral load undetectability/treatment status [for men living with HIV]).	Among HIV-negative/unknown men, those in the Common Drugs class (compared to Limited Drugs class) were more likely to: Know ART can reduce viral load and prevention transmission (aOR: 1.24; 95% CI: 1.11, 1.40) Know PEP can be taken after sex to reduce risk of HIV (aOR:1.16; 95% CI: 1.04, 1.30) Know PreP can prevent HIV acquisition (aOR: 1.24; 95% CI: 1.11, 1.38) Be interested in taking PrEP (aOR: 1.30; 95% CI: 1.16, 1.44) No significant associations between Common Drug class and Limited Drug class among men with HIV and knowledge of ART, PEP, or PrEP or viral load/ART adherence.
Carney et al. [57]	2023	Sexual health variables (HIV testing, diagnosis of STI, PrEP awareness and use)	Did not explicitly measure co-use, used LCA. *LCA profiles:* Depressant and Stimulant Use High Polysubstance Use Low Substance Use with Moderate Cannabis Use High Cannabis, Stimulant and Alcohol Use	LCA profiles based on substance use, including: *Alcohol*: High-risk alcohol use (AUDIT) *Cannabis*: ASSIST-C, any nonmedical cannabis use past 3 months	Bivariate multinomial logistic regression used to calculate odds ratios for correlates of latent class membership.	Participants in the High Polysubstance Use class were less likely to ever test for HIV than those in the Low Substance Use with Moderate Cannabis Use class (aOR: 0.21; 95% CI: 0.05, 0.93). People in High Cannabis, Stimulant and Alcohol Use were significantly more likely to have repeat HIV testing and STI diagnosis compared to the Low Substance Use with Moderate Cannabis Use class.
Carter et al. [54]	2018	*HIV risk:*Self-report adherence to cART, measured with three levels: never/not currently on cART, < 95% (non-adherent), > 95% (adherent)	Did not explicitly measure co-use, used LCA. *Relevant LCA profile:* Socially Acceptable Polysubstance Users: includes tobacco, alcohol, cannabis use	LCA profiles based on substance use, including: *Alcohol*: Past-year alcohol use *Cannabis*: Past-year cannabis use	LCA to identify substance use profiles. Multinomial logistic regression to examine predictors of latent class membership.	Women with suboptimal ART adherence had higher odds of Socially Acceptable Polysubstance Use compared to people adherent (> 95%) (aOR: 2.12; 95% CI: 1.23, 3.65). Women never/not currently on cART had lower odds of Socially Acceptable Polysubstace Use (aOR: 0.38; 95% CI: 0.21, 0.68) compared to optimal adherence.
Choi et al. [41]	2022	*Cannabis*: Frequency of cannabis use (high frequency defined as > 10 days/mo) *Alcohol* Unhealthy alcohol use (> 8 on the AUDIT scale)	*Concurrent use:* High concurrent use, categorization of both unhealthy alcohol use and frequency of cannabis use	Biologically-confirmed STI, multiple sex partners, recent transactional sex, lower condom intentions	Logistic models calculated to assess the relationship between high concurrent use and diverse adverse health outcomes	High concurrent use associated with: biologically confirmed STI (aOR: 2.38; 95% CI: 1.52, 3.72), risky sexual behaviors, such as multiple sex partners (aOR: 2.76; 95% CI: 1.73, 4.39), recent transactional sex (aOR: 4.54; 95% CI: 2.60, 7.94), and lower condom intentions (aOR: 2.20; 95% CI: 1.41, 3.43).
Chung et al. [61]	2022	LCA profiles based on: *Alcohol:* Self-report of use and perceived frequency of partner alcohol use in the past year from ages 18–20, ranging from none through daily. *Cannabis:*Same as alcohol. *HIV risk:* Sexual risk behavior (SRB) score (range: 0–3) assessed via the Adolescent Sexual Activity Index, which includes the items: sexual intercourse with 2 partners in the past year; did not ‘always’ use birth control; and did not use condoms. STIs in past-year measured through self-report (inclusive of chlamydia, herpes, HIV, genital warts, or other).	Did not explicitly measure co-use, used LCA. *Relevant LCA profiles:* Co-Use: Occasional Alcohol and Cannabis Use: represented women who reported relatively low alcohol and cannabis use, and partners’ slightly higher alcohol and cannabis than their own. Co-Use: Increasing Alcohol and Occasional Cannabis Use: an increase from monthly (at age 18) to weekly (age 20) alcohol use and relatively low cannabis use, with partner alcohol use increasing to weekly drinking at age 20 and similar cannabis use. Co-Use: Frequent Cannabis and Occasional Alcohol Use: cannabis use 2–3 times/week and less than monthly alcohol use	NA	LCA to identify profiles of women’s and perceived partners past-year alcohol and cannabis use. The LCA model included 18 indicators, including women’s past year alcohol and cannabis use, perceived partner alcohol and cannabis use, sex behavior scores, and report of past year STI.	Across all three LCA profiles, SRB score generally increased from ages 18–20. At age 20, there was no significant difference in SRB score across the 3 profiles. At ages 18 and 19, women in the “Co-Use: Increasing Alcohol” profile were less likely to report a past-year STI diagnosis compared to both the “Co-Use: Frequent Cannabis” and “Co-Use: Occasional” profiles. At age 20, there was no difference between profiles in likelihood of reporting a past-year STI.
D’Anna et al. [39]	2021	*Alcohol*: 10-item AUDIT scale (denoting harmful drinking) *Cannabis*: ASSIST-M to measure problem marijuana use	Interaction effects of problem marijuana and problem alcohol use	Four measures of sex behaviors in the past 3 months: (1) unprotected sex for money, drugs, or other things; (2) unprotected sex with someone who has/suspected to have HIV/or an STI; (3) unprotected sex with a partner you knew or suspected of using injection drugs; and (d) unprotected sex while under the influence of a substance	Logistic regression	*Main effects:* Problem cannabis use and risky sexual behavior was significant, even when controlling for problematic alcohol use. Problem alcohol use and risky sexual behavior was significant. *Interaction:* The interaction term for problem cannabis use and alcohol was not significant.
Degarege et al. [43]	2022	Various combinations of polysubstance use, including alcohol and marijuana *Alcohol*: Harmful and hazardous drinking (AUDIT scores≥8) *Cannabis*: Active use of cannabis, use within past 48 h verified with a urine drug screen	Polysubstance use was determined by adding the use of each substance including harmful/hazardous alcohol consumption, marijuana, cocaine/crack, amphetamine/methamphetamine, opiate, and tobacco.	ART adherence (how many doses missed in the last four days)	Poisson regression model was used to assess the relationship of substance use with the number of doses missed over the prior four days.	The mean number of doses missed over the past four days was greater among participants who were using: alcohol and marijuana (B: 0.73, 95% CI 0.05, 1.41, *p* = 0.036), and alcohol, cocaine, marijuana, and tobacco (B: 0.97, 95% CI 0.25, 1.70, *P* = 0.009).
El-Bassel et al. [58]	2019	*Sexual behaviors:* How many sex partners in the past year, how many times they had unprotected sex, diagnosis of STI in past year, HIV status	Did not explicitly measure co-use, used LCA. *LCA profiles:* Polydrug Users Mild Polydrug Users, with Severe Alcohol and Marijuana Use Alcohol and Marijuana Users	LCA profiles based on 11 dichotomous variables measuring substance use, including: *Alcohol*: Alcohol use and misuse (defined as binge drinking, 5 + alcoholic beverages on one occasion) in the past 90 days *Cannabis*: Use of cannabis in past 90 days	Used binomial logistic regression to examine predictors of drug use typology.	Men in the Mild Polydrug Users, with Severe Alcohol and Marijuana Use class: More likely to have multiple sexual partners when compared to Polydrug Users and Alcohol and Marijuana Users classes. More likely to have a higher number of unprotected sexual partners compared to the Alcohol and Marijuana Users class. More likely to have HIV compared to Alcohol and Marijuana Users class. Women in the Polydrug Users class: More likely to have greater number of unprotected sexual partners compared to Mild Polydrug Users, with Severe Alcohol and Marijuana Use class and Alcohol and Marijuana Users classes More likely to have any form of STI compared to Alcohol and Marijuana Users class Women in the Mild Polydrug Users, with Severe Alcohol and Marijuana Use class: Higher rates of self-reported HIV when compared to the Alcohol and Marijuana Users class
Firkey et al. [42]	2021	Day-level substance use, including: *Alcohol*: Timeline followback assessed the number of standard drinks of alcohol consumed on each day *Cannabis*: Use (yes or no) for each day during the timeline followback	Alcohol and cannabis co-use (yes) if both occurred on same day	Condomless anal sex	GEE models to examine participant substance use and condomless anal sex with a partner of any HIV status (or HIV- or unknown HIV status).	The odds of engaging in CAS were significantly higher for sexual events in which the participant reported cannabis and alcohol co-use (aOR: 2.98; 95% CI: 1.27, 6.97) compared to events when neither substance use. No significant difference when compared to either substance used alone. No significant difference in sex with negative/unknown HIV status partner when engaging in alcohol and cannabis co-use compared to no substance use or either substance used alone.
Green et al. [64]	2017	LCA profiles based on substance use, including: *Alcohol*: Past-year frequency of alcohol use for grades 8–12 was collected on a 7-point scale *Cannabis*: Past-year frequency of cannabis use for grades 8–12	Did not explicitly measure co-use, used LCA profiles. *LCA profiles:* Non-Use Moderate Alcohol: moderate and increasing levels of alcohol, little to no marijuana use Moderate Alcohol/Increasing Marijuana: increasing levels of marijuana use across high school, similar levels of alcohol High Dual Use: high alcohol and marijuana use across high school	Sexual outcomes: multiple sexual partners, sex without a condom (past month), teenage pregnancy, and contraction of an STI	Constructed longitudinal latent profile analysis to model frequency of alcohol and marijuana use from grade 8–12. Did pairwise comparisons to look at outcomes across alcohol and marijuana classes.	The Moderate Alcohol/Increasing Marijuana class: Had a higher frequency of sex without a condom than the Non-Use class. Highest proportion of multiple sex partners compared to all classes (46%), followed by High Dual Use (39%) Higher proportion of STI diagnosis (24%) compared to Moderate Alcohol (22%) and Non-Use (11%) class, but lower than High Dual Use (30%)
Houck et al. [62]	2006	Cluster analysis based on five factors, including: *Alcohol*: How many days of the past 30 days participants drank alcohol. *Cannabis*: How many days of the past 30 they used any form of cannabis *Co-use:* Combined score (0–60) of how many days participants used alcohol and marijuana *Sexual risk behaviors:* Number of unprotected sex acts (calculated from number of sex acts and total number of times participants reported using condoms during those acts (asked for each partner) in the past 90 days)	Did not explicitly measure co-use, used cluster analysis. *Relevant cluster*: Cluster 2: alcohol and marijuana use/unprotected sex	NA	Cluster analysis to group participants on similarity for five factors (i.e., unprotected sex, alcohol/marijuana use, drug use, mental health, arrest/dropout). ANOVA and chi-squared to examine differences by cluster membership.	Among men: no significant differences by cluster for presence of STI, number of sexual partners in past 90 days, or being with a partner with HIV/STI risks Among women: no significant differences by cluster for presence of STI. Women in the lower risk group (Class 3) reported significantly fewer partners in the last 90 days than the other clusters (including Cluster 2). Women in Cluster 2 were significantly more likely than those in the other clusters to report having been with a partner with HIV/STI risks.
Ma et al. [46]	2025	Polysubstance use, including: *Alcohol*: AUDIT-C to assess alcohol use severity in the past year *Cannabis*: ASSIST score in the past 3 months	Reporting use of ≥ 2 substances. Also measured overall number of substances used (0–5) and number of illicit drugs used (0–3).	Past 30-day self-reported ART adherence	Estimated the relationship between time-varying substance use severity and ART adherence using linear mixed models.	Number of substances used was associated with 0.6% lower ART adherence (95% CI: −0.9%, −0.3%) for each additional substance reported. When categorizing the number of substances use, ≥ 3 substances showed a greater decrease in ART adherence compared to fewer substances used.
Przybyla et al. [47]	2022	Polysubstance use, including: *Alcohol:* Yes/no current alcohol use *Cannabis:* Yes/no current cannabis use	Current use of two or more substances (i.e., alcohol, cannabis, heroin, cocaine, methamphetamine, heroin, and hallucinogen)	ART adherence (any reported missed doses of ART in the last seven day)	Multivariate logistic regression to examine the relationship between different substance use categories (i.e., one substance, polysubstance) and ART adherence.	Polysubstance use (aOR: 0.46; 95% CI: 0.22, 0.96) associated with decreased odds of being adherent compared to no substance use. Similar results for one substance (aOR: 0.45; 95% CI: 0.23, 0.89) compared to no substance use.
Reynolds et al. [65]	2019	LCA profiles based on 15 substance use variables, including: *Alcohol*: Past-month alcohol use or use immediately before or during sex *Cannabis*: Past-month cannabis use or use immediately before or during sex	Did not explicitly measure co-use, used LCA. *LCA profiles:* Abstainers Amphetamine Alcohol and Marijuana No Sex-with-Alcohol Poly Drug	Sex behaviors (past-30 days for vaginal or anal intercourse, oral sex, HIV/STI positivity)	Estimated the relationship between the assigned class membership and outcomes of interest with multinomial logistic regression.	Compared to the Abstinent reference group, only the Alcohol and Marijuana group had a higher odds of vaginal intercourse (OR: 2.48; 95% CI: 1.22, 5.05), while the Poly Drug group was significantly less likely to report vaginal intercourse, but more likely to engage in anal intercourse. No significant differences across latent classes in HIV positivity.
Ritchwood et al. [40]	2016	*Alcohol*: Incorporated measures of frequency to create an 8-level variable: used alcohol never, sometime, once in past year, more than once in past year, in past month, more than once in past month, used in past week, and used more than once in past week. *Cannabis*: Same operationalization as alcohol.	Interaction effect between alcohol and cannabis	Sex behaviors included four variables: number of sex partners, frequency of condom use during the last 90 days, condom use at last intercourse	Multilevel modelling to test whether substance use predicts risky sexual behavior	The simultaneous use of alcohol and marijuana create an antagonizing (expected increase in risky sexual behaviors is beyond what we would expect by summing individual effects) effect (within-groups: B ≤=0.118, *p* < 0.001; between-groups: B <=0.236, *p* < 0.05)
Rodriguez-Bolanos et al. [67]	2022	LCA profiles based on: *Alcohol*: Alcohol use in the last 30 days; binge drinking if> 5 drinks on one occasion *Cannabis*: Cannabis use in the past 3 months	Did not explicitly measure co-use, used LCA. *LCA profiles:* Class 1: low substance use Class 2: high alcohol use Class 3: polysubstance use, high use of all substances including alcohol, marijuana, sex drugs, stimulants, and depressants	Sex behaviors, including condomless anal sex in the past 3 months, or serosorting	Multinomial logistic regression to identify differences in membership in each class	Compared to Class 1, membership in Class 3 was more likely among MSM if they engaged in either CAS (aOR: 2.92; 95% CI: 1.30, 3.12) or serosorting (aOR: 2.02; 95% CI: 2.02, 4.22).
Rosen et al. [52]	2024	LCA profiles based on self-reported measured of substance use, including: *Alcohol*: AUDIT-C (2 items), with scores > 3 defined as heavy alcohol use. *Cannabis*: Cannabis use in the past 30 days	Did not explicitly measure co-use, used LCA. *LCA profiles:* Heavy Alcohol Use Only Cannabis, Crack & Heavy Alcohol Use Whoonga & Crack Use	HIV viremia, defined using a cutpoint of ≥ 50 RNA copies/mL	Multivariable mixture model to determine association between LCA classes and HIV viremia	Relative to the Heavy Alcohol Use Only class, the Cannabis, Heavy Alcohol, & Crack Use class had higher probabilities of HIV viremia (*p* < 0.001). In multivariable analysis, HIV viremia did not differ between these two classes (aOR: 1.17; 95% CI: 0.74, 1.86).
Shrader et al. [48]	2024	Substance use during sex, including: *alcohol*, *cannabis*, ecstasy, cocaine/crack, poppers, methamphetamine, psychedelics or party drugs, prescription painkillers	Sex polydrug use defined as the use of 2 or more substances (or 1 substance and alcohol).	Condomless sex	Multilevel logistic regression models to assess the relationship between respondents and sexual partner-level variables, and bipartite network analyses.	Respondents had higher odds of condomless sex with sex-polydrug use (OR: 1.48; 95% CI: 1.02, 2.14). Statistically significant correlation between cannabis and alcohol of respondents and sex partners used in tandem during sex.
Tobin et al. [63]	2016	LCA profiles based on 6 substances, including: *Alcohol*: Heavy alcohol use (drinking 3 + drinks in a typical day) in the past 90 days, and binge drinking (> 6 drinks) in the past 90 days *Cannabis*: Cannabis use in the past 90 days	Did not explicitly measure co-use, used LCA. *LCA profiles:* Light Users Poly-Users Alcohol/Marijuana: high probability of heavy alcohol use, binge drinking, and marijuana use	Sex behaviors (sex under the influence, having an STI in past 90 days, frequency of condom use, frequency of sex)	LCA to define subgroups of substance use. Bivariate and multivariate analysis.	Alcohol/Marijuana class had higher relative risk of having sex under the influence (RRR = 2.87; 95% CI: 1.27, 11.5) compared to Light User class. Poly-User class had higher relative risk of having an STI in the prior 90 days compared to Alcohol/Marijuana class (RRR: 5.32; 95% CI: 1.52–18.6).
Tsuyuki et al. [49]	2019	Polysubstance use measure based on: *Alcohol*: Alcohol misuse measured with AUDIT 10-item scale *Cannabis*: Number of days that marijuana and hashish were reported in prior 3 months	Polysubstance use: total number of substances reported in the past 3 months (yes/no), including stimulants, *cannabis*, and *alcohol* misuse	Viral load, ART adherence, ART use	Generalized linear mixed models (GLMMs) with logit link function to estimate the odds of substance use on having an undetectable HIV viral load	The number of substances used was significantly associated with decreased odds of taking ARTs in the past 3 months among Brazilian heterosexual men (aOR:0.25; 95% CI: 0.08, 0.78). Number of substances not associated with undetectable viral load among Thai or Brazilian men.
Wechsberg et al. [66]	2012	LCA profiles based on: *Alcohol*: Frequent drinking: two or more drinking episodes per week Heavy drinking: seven or more drinks per day *Drug use:* Self-reported use of cannabis, methamphetamine, heroin, and Mandrax (methaqualone) in the last month	Did not explicitly measure co-use, used LCA. *Relevant LCA profiles:* Marijuana and Alcohol High Alcohol and Other Drug Polydrug	Past-month unprotected sex with main sex partner, unprotected sex with sex partners other than main partner, alcohol or drug-impairment during last sex act	LCA to construct measure of polysubstance use, using the alcohol and other drug use indicators. Multivariate logistic regression to examine the impact of class membership on sex risk outcomes.	Women in the Marijuana and Alcohol class had lower odds of reporting past month unprotected sex with their main sex partner compared with women in the Polydrug Use class (aOR: 0.58; 95% CI: 0.36–0.94). Women in the High Alcohol and Other Drug class had higher odds of alcohol or other drug impairment during last sex act compared with women in the Marijuana and Alcohol class (aOR: 1.99; 95% CI: 1.21–3.28).
Wiginton et al. [53]	2024	LCA profiles based on 9 substance use and sociodemographic indicators, including: *Alcohol*: Alcohol, Smoking and Substance Involvement Screening Test (score ≥ 11 for alcohol items) *Cannabis*: Alcohol, Smoking and Substance Involvement Screening Test (score > 4 for cannabis items), global measure of misuse of illicit drugs because specific drug misuse was low	Did not explicitly measure co-use, used LCA. *Relevant LCA profiles:* Polydrug-Socioeconomic Syndemic: high probabilities of alcohol and marijuana misuse, moderate probabilities of illicit drug misuse, food and housing insecurity, and legal involvement Distress-Socioeconomic Syndemic Syndemic-free	HIV care engagement (based on number of missed appointments), HIV treatment adherence (based on how many missed doses in the last 30 days), and HIV viral load	Assessed class differences in outcomes with chi-square tests.	Having missed or had no HIV care appointments in the past year was higher in the “Polydrug-Socioeconomic Syndemic” class than the other two classes. Having missed any HIV treatment dose in the past month was higher in the Polydrug-Socioeconomic class than the Syndemic-free class. No differences between classes in regard to unsuppressed HIV viral load prevalence.

## Data Availability

No datasets were generated or analysed during the current study.
