# Supplementary material for: Alcohol and Cannabis co-use and HIV risk, Treatment and Prevention Outcomes: A Scoping Review
Source: Curr Addict Rep. Author manuscript; Available in PMC 2026 May 21. (PMC13188471; doi:10.1007/s40429-025-00707-x)
Supplement: Supplementary tables [file NIHMS2175574-supplement-Supplementary_tables.docx]

**Supplemental Table 1. Search Syntax for All Databases**

| **Database** | **Syntax** |
| --- | --- |
| **Medline (OVID)** | 1. (cannabi* OR marijuana OR marihuana OR CBD OR THC OR dronabinol OR tetrahydrocannabinol OR delta-9 OR "delta 9" OR delta-8 OR "delta 8" OR hemp OR hash* OR pot OR wax OR kief).mp.  2. exp cannabinoids/ OR exp cannabis/ OR exp "Marijuana Use"/ OR exp "Marijuana Abuse"/  3. 1 OR 2  4. (HIV OR "human immunodeficiency virus" OR AIDS OR "acquired immunodeficiency syndrome" OR PrEP OR "pre-exposure prophylaxis" OR ARV OR antiretroviral OR "anti-retroviral" OR "anti-retrovirus" OR "anti retrovirus" OR "viral suppress*" OR seroconvers*).mp.  5. exp "Pre-Exposure Prophylaxis"/ OR exp "Acquired Immunodeficiency Syndrome"/ OR exp "HIV"/ OR exp "Antiretroviral Therapy, Highly Active"/  6. 4 OR 5  7. (alcohol* OR ethanol* OR drink*).mp.  8. exp "Alcohol Drinking"/ OR exp "Alcohol-Related Disorders"/ AND exp "Ethanol"/ OR exp "Alcoholic Beverages"/  9. 7 OR 8  10. (concurr* OR “co-use” OR “co use” OR complement* OR substitut* OR simult* OR dual* OR poly*).mp.  11. 3 AND 6 AND 9 AND 10   12. Limit 11 to English Language |
| **APA PsycInfo (OVID)** | 1. (cannabi* OR marijuana OR marihuana OR CBD OR THC OR dronabinol OR tetrahydrocannabinol OR delta-9 OR "delta 9" OR delta-8 OR "delta 8" OR hemp OR hash* OR pot OR wax OR kief).mp.  2. "cannabis use"/ or "cannabis use disorder"/ or exp cannabis/  3. 1 OR 2  4. (HIV OR "human immunodeficiency virus" OR AIDS OR "acquired immunodeficiency syndrome" OR PrEP OR "pre-exposure prophylaxis" OR ARV OR antiretroviral OR "anti-retroviral" OR "anti-retrovirus" OR "anti retrovirus" OR "viral suppress*" OR seroconvers*).mp.  5. exp Pre-Exposure Prophylaxis/ or exp HIV/ or exp Antiretroviral Drugs/  6. 4 OR 5  7. (alcohol* OR ethanol* OR drink*).mp.  8. exp "alcohol use"/ or exp "alcohol use disorder"/ or exp Alcoholic Beverages/ or exp ethanol/  9. 7 OR 8  10. (concurr* OR "co-use" OR "co use" OR complement* OR substitut* OR simult* OR dual* OR poly*).mp.  11. exp polydrug abuse/  12. 10 OR 11  13. 3 AND 6 AND 9 AND 12  14. Limit 13 to English Language |
| **Web of Science** | 1. TI=(cannabi* OR marijuana OR marihuana OR CBD OR THC OR dronabinol OR tetrahydrocannabinol OR delta-9 OR "delta 9" OR delta-8 OR "delta 8" OR hemp OR hash* OR pot OR wax OR kief) OR AB=(cannabi* OR marijuana OR marihuana OR CBD OR THC OR dronabinol OR tetrahydrocannabinol OR delta-9 OR "delta 9" OR delta-8 OR "delta 8" OR hemp OR hash* OR pot OR wax OR kief) OR KP=(cannabi* OR marijuana OR marihuana OR CBD OR THC OR dronabinol OR tetrahydrocannabinol OR delta-9 OR "delta 9" OR delta-8 OR "delta 8" OR hemp OR hash* OR pot OR wax OR kief)  2. TI=(HIV OR "human immunodeficiency virus" OR AIDS OR "acquired immunodeficiency syndrome" OR PrEP OR "pre-exposure prophylaxis" OR ARV OR antiretroviral OR "anti-retroviral" OR "anti-retrovirus" OR "anti retrovirus" OR "viral suppress*" OR seroconvers*) OR AB=(HIV OR "human immunodeficiency virus" OR AIDS OR "acquired immunodeficiency syndrome" OR PrEP OR "pre-exposure prophylaxis" OR ARV OR antiretroviral OR "anti-retroviral" OR "anti-retrovirus" OR "anti retrovirus" OR "viral suppress*" OR seroconvers*) OR KP=(HIV OR "human immunodeficiency virus" OR AIDS OR "acquired immunodeficiency syndrome" OR PrEP OR "pre-exposure prophylaxis" OR ARV OR antiretroviral OR "anti-retroviral" OR "anti-retrovirus" OR "anti retrovirus" OR "viral suppress*" OR seroconvers*)  3. TI=(alcohol* OR ethanol* OR drink*) OR AB=(alcohol* OR ethanol* OR drink*) OR KP=(alcohol* OR ethanol* OR drink*)  4. TI=(concurr* OR "co use" OR "co-use" OR complement* OR substitut* OR simult* OR dual* OR poly*) OR AB=(concurr* OR "co use" OR "co-use" OR complement* OR substitut* OR simult* OR dual* OR poly*) OR KP=(concurr* OR "co use" OR "co-use" OR complement* OR substitut* OR simult* OR dual* OR poly*)  5. 1 AND 2 AND 3 AND 4  6. Filter 5 by English Language |
| **Embase** | 1. (cannabi* OR marijuana OR marihuana OR CBD OR THC OR dronabinol OR tetrahydrocannabinol OR delta-9 OR "delta 9" OR delta-8 OR "delta 8" OR hemp OR hash* OR pot OR wax OR kief):ti,ab,kw  2. 'cannabinoid'/syn OR 'cannabis use'/syn OR 'cannabis addiction'/syn  3. #1 OR #2  4. (HIV OR "human immunodeficiency virus" OR AIDS OR "acquired immunodeficiency syndrome" OR PrEP OR "pre-exposure prophylaxis" OR ARV OR antiretroviral OR "anti-retroviral" OR "anti-retrovirus" OR "anti retrovirus" OR "viral suppress*" OR seroconvers*):ti,ab,kw  5. 'pre-exposure prophylaxis'/syn OR 'acquired immune deficiency syndrome'/syn OR 'Human immunodeficiency virus'/syn OR 'highly active antiretroviral therapy'/syn  6. #4 OR #5  7.(alcohol* OR ethanol* OR drink*):ti,ab,kw  8. 'drinking behavior'/syn OR 'alcoholism'/syn OR 'alcohol'/syn OR 'alcoholic beverage'/syn  9. #7 OR #8  10. (concurr* OR (co NEAR/1 use) OR complement* OR substitut* OR simult* OR dual* OR poly*):ti,ab,kw  11. 'polysubstance use'/syn  12. #10 OR #11  13. #3 AND #6 AND #9 AND #12  14. Filter #13 by English Language |
| **CINAHL (EBSCOHost)** | 1. TI(cannabi* OR marijuana OR marihuana OR CBD OR THC OR dronabinol OR tetrahydrocannabinol OR delta-9 OR "delta 9" OR delta-8 OR "delta 8" OR hemp OR hash* OR pot OR wax OR kief) OR AB(cannabi* OR marijuana OR marihuana OR CBD OR THC OR dronabinol OR tetrahydrocannabinol OR delta-9 OR "delta 9" OR delta-8 OR "delta 8" OR hemp OR hash* OR pot OR wax OR kief)   2. (MH "Cannabis+")   3. 1 OR 2  4. TI(HIV OR "human immunodeficiency virus" OR AIDS OR "acquired immunodeficiency syndrome" OR PrEP OR "pre-exposure prophylaxis" OR ARV OR antiretroviral OR "anti-retroviral" OR "anti-retrovirus" OR "anti retrovirus" OR "viral suppress*" OR seroconvers*) OR AB(HIV OR "human immunodeficiency virus" OR AIDS OR "acquired immunodeficiency syndrome" OR PrEP OR "pre-exposure prophylaxis" OR ARV OR antiretroviral OR "anti-retroviral" OR "anti-retrovirus" OR "anti retrovirus" OR "viral suppress*" OR seroconvers*)  5. (MH "Human Immunodeficiency Virus+") OR (MH "HIV Infections+") OR (MH "HIV-Positive Persons+") OR (MH "Pre-Exposure Prophylaxis") OR (MH "Anti-Retroviral Agents+")  6. 4 OR 5   7. TI(alcohol* OR ethanol* OR drink*) OR AB(alcohol* OR ethanol* OR drink*)  8.(MH "Alcohol Drinking+") OR (MH "Alcohol-Related Disorders+") OR (MH "Ethanol+")  9. 6 OR 7  10. TI(concurr* OR ("co use") OR ("co-use") OR complement* OR substitut* OR simult* OR dual* OR poly*) OR AB(concurr* OR ("co use") OR ("co-use") OR complement* OR substitut* OR simult* OR dual* OR poly*)  11. 3 AND 6 AND 9 AND 10  11. Filter #11 by English Language |
| **Scopus** | 1. TITLE-ABS-KEY(cannabi* OR marijuana OR marihuana OR CBD OR THC OR dronabinol OR tetrahydrocannabinol OR delta-9 OR "delta 9" OR delta-8 OR "delta 8" OR hemp OR hash* OR pot OR wax OR kief)  2. TITLE-ABS-KEY(HIV OR "human immunodeficiency virus" OR AIDS OR "acquired immunodeficiency syndrome" OR PrEP OR "pre-exposure prophylaxis" OR ARV OR antiretroviral OR "anti-retroviral" OR "anti-retrovirus" OR "anti retrovirus" OR "viral suppress*" OR seroconvers*)  3.TITLE-ABS-KEY(alcohol* OR ethanol* OR drink*)  4. TITLE-ABS-KEY(concurr* OR (co W/1 use) OR complement* OR substitut* OR simult* OR dual* OR poly*)  5. #1 AND #2 AND #3 AND #4  6. Filter #5 by English Language |
| **PubMed** | 1. cannabi*[tiab] OR marijuana[tiab] OR marihuana[tiab] OR CBD[tiab] OR THC[tiab] OR dronabinol[tiab] OR tetrahydrocannabinol[tiab] OR delta-9[tiab] OR "delta 9"[tiab] OR delta-8[tiab] OR "delta 8"[tiab] OR hemp[tiab] OR hash*[tiab] OR pot[tiab] OR wax[tiab] OR kief[tiab]  2. "Cannabinoids"[Mesh] OR "Cannabis"[Mesh] OR "Marijuana Use"[Mesh] OR "Marijuana Abuse"[Mesh]  3. #1 OR #2  4. HIV[tiab] OR "human immunodeficiency virus"[tiab] OR AIDS[tiab] OR "acquired immunodeficiency syndrome"[tiab] OR PrEP[tiab] OR "pre-exposure prophylaxis"[tiab] OR ARV[tiab] OR antiretroviral[tiab] OR "anti-retroviral"[tiab] OR "anti-retrovirus"[tiab] OR "anti retrovirus"[tiab] OR "viral suppress*"[tiab] OR seroconvers*[tiab]  5. "Pre-Exposure Prophylaxis"[Mesh] OR "Acquired Immunodeficiency Syndrome"[Mesh] OR "HIV"[Mesh] OR "Antiretroviral Therapy, Highly Active"[Mesh]  6. #4 OR #5  7. alcohol*[tiab] OR ethanol*[tiab] OR drink*[tiab]   8. "Alcohol Drinking"[Mesh] OR "Alcohol-Related Disorders"[Mesh] AND "Ethanol"[Mesh] OR "Alcoholic Beverages"[Mesh]  9. #7 OR #8  10.concurr*[tiab] OR “co use”[tiab~1] OR “co-use” OR complement*[tiab] OR substitut*[tiab] OR simult*[tiab] OR dual*[tiab] OR poly*[tiab]  11. #3 AND #6 AND #9 AND #10  12. Filter #11 by English Language |
